# Detecting Alzheimer’s Disease Using Ocular Tissue and Imaging: What Do We Know?

**DOI:** 10.3390/biom15111519

**Published:** 2025-10-28

**Authors:** Minali Prasad, Manju L. Subramanian

**Affiliations:** 1 Boston University Chobanian & Avedisian School of Medicine, Boston, MA 02118, USA; 2 Department of Ophthalmology and Visual Science, Boston Medical Center, Boston, MA 02118, USA

**Keywords:** Alzheimer’s disease, ocular imaging, ocular fluids

## Abstract

Alzheimer’s disease (AD) is a progressive neurodegenerative condition with increasing global prevalence. As early diagnosis becomes critical for timely symptomatic management, noninvasive and easily accessible biomarkers are needed. Given the shared embryologic origins between the eye and the brain, ocular imaging has emerged as a promising diagnostic technique. This review summarizes the associations between AD, ocular imaging and fluid biomarkers in the anterior and posterior segment. We also describe the underlying pathophysiology that explains the connections between each ocular structure and the brain in the context of AD. Optical coherence tomography (OCT), OCT angiography, and fundus photography are the most common imaging modalities utilized in AD research. However, these techniques may or may not be feasible in primary care or neurologic clinical settings. Compared to plasma biomarker analysis, which is minimally invasive and nearing clinical implementation, ocular biomarkers remain primarily valuable in research investigations.

## 1. Introduction

Alzheimer’s disease (AD) is the most common type of dementia worldwide, with a projected global prevalence of 131.5 million by 2050 [1]. It is currently the seventh leading cause of death in the United States, with no known cure [2]. The earliest symptom of AD is typically short-term memory loss, often followed by progressive decline in executive functioning, language, and visuospatial skills as well as neuropsychiatric symptoms in moderate to late stages [2].

The pathogenesis of AD is characterized by the loss of cholinergic neurons and the extracellular accumulation of amyloid-β (Aβ) peptide resulting from the impaired degradation of amyloid precursor protein (APP) [2]. Histopathological examination of the brain in AD demonstrates hallmark features such as neuritic plaques (extracellular Aβ deposits), intracellular neurofibrillary tangles of hyperphosphorylated tau (pTau), and widespread cortical neuronal degeneration [2]. These neuropathological changes can begin decades before the onset of clinical symptoms, which often limits the effectiveness of treatment initiated later in the disease course. Diagnosis is primarily based on clinical evaluation, functional assessment, and neuropsychological testing. Confirmation of AD pathology can be achieved with fluorodeoxyglucose–positron emission technology and cerebrospinal fluid (CSF) analysis [2]. However, these diagnostic techniques are invasive, costly, and largely confined to academic health centers and research settings. This highlights the urgent need for alternative diagnostic approaches that can enable earlier detection and therapeutic intervention.

Given that the eye and the brain share a common embryological origin [3,4,5], neurodegenerative disease processes associated with AD may be observed in ocular tissue [6]. This narrative review aims to explore the role of ocular protein biomarkers and imaging in the detection of AD. We examine structures within both the anterior and posterior segments of the eye, as well as ocular fluids including tear secretions, aqueous humor, and vitreous humor.

### Ocular Anatomy and Function

**Figure 1:**
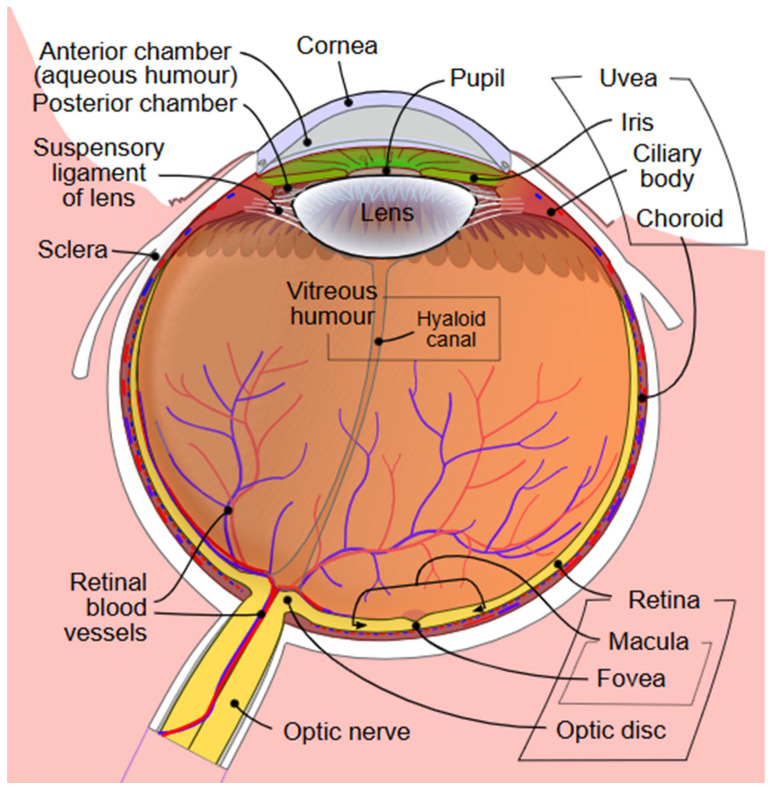
Diagram of the anterior and posterior chambers of the eye [20].

Figure 1 demonstrates the structures of the eye. The conjunctiva is a thin, vascularized mucous membrane that lines the eyelids and globe, and functions to protect the ocular surface, support immune surveillance, and produce mucin for the tear film for lubrication and barrier function [7]. The cornea provides the majority of the eye’s refractive power while maintaining a transparent, avascular barrier [8]. The cornea is comprised of five layers, including an epithelium with tight junctions, Bowman’s layer, stroma, Descemet’s membrane, and endothelium [8]. These layers regulate hydration and block pathogens [8]. The cornea is heavily innervated, leading to blink reflexes, wound healing, and tear film production [9]. The ocular surface is coated by a tear fluid which is made of secretions containing lipids, proteins, and electrolytes from the lacrimal gland, meibomian gland, and conjunctival goblet cells [10]. This tear film lubricates, nourishes, and protects the surface, which can influence vision quality [10].

The iris is a muscle that modulates the pupillary diameter via sphincter and dilator muscles, as well as signals from the autonomic nervous system, and helps reduce optical aberrations [11,12,13]. Behind the iris, the crystalline lens is a transparent, avascular structure composed of fiber cells surrounded by a capsule [14]. The lens is responsible for refraction and accommodation [14]. These structures, from the cornea to the lens, form the anterior chamber of the eye, which is full of aqueous humor, a fluid secreted by the ciliary body [15]. This fluid exits the eye via the trabecular meshwork–Schlemm’s canal pathway and the uveoscleral pathway [15]. Decreased fluid drainage can cause irreversible vision loss in glaucoma [15].

The posterior chamber (the space between the lens and retina) is composed of vitreous humor, a collagenous substance that provides mechanical support for the globe [16]. Over time, this fluid liquefies and the collagen network aggregates, predisposing aging individuals to posterior vitreous detachment and other retinal complications [16]. The retina converts photons of light to neural signals via phototransduction in rod and cone photoreceptor cells [17]. The retina has other specialized cells, including bipolar, horizontal, amacrine, and ganglion cells, for detecting contrast and motion [17]. The neural signals are transmitted to the retinal ganglion cell axons which come together to form the optic nerve [18]. The optic nerve is connected to areas of the brain including the lateral geniculate nucleus in the thalamus and the superior colliculus in the midbrain [18].

The choroid is deep to the retina and composed of the choriocapillaris, Sattler’s and Haller’s layers, Bruch’s membrane, and the suprachoroid [19]. The choroid provides blood flow to the outer retina and retinal pigment epithelium, and removes metabolic waste from the photoreceptor cells [19].

Table 1 summarizes the associations between each ocular structure and AD that will be discussed in this review.

## 2. Anterior Segment

### 2.1. Conjunctiva

To our knowledge, there exists one report regarding the association between conjunctival microcirculation and AD. Smith et al. compared vascular parameters of the conjunctival microcirculation between patients with and without AD using computer-assisted intravital microscopy, and found that participants with AD had a significantly higher whole blood viscosity and greater severity of vasculopathy [21]. This suggests that AD pathology is associated with microvascular abnormalities in the external eye, though the underlying mechanism and temporal relationship remain unclear.

### 2.2. Cornea

Nerve growth factor is responsible for nourishing cholinergic neurons in the basal forebrain. In the pathogenesis of AD, the degeneration of cholinergic neurons is accompanied by decreases in nerve growth factor. The cornea contains high levels of both nerve growth factor and acetylcholine, so neuronal degeneration in the forebrain may also be reflected in the cornea, leading to changes in corneal nerve fibers [22,23,24]. In fact, corneal changes in sensitivity and morphology have been associated with the presence of AD, and proposed as biomarkers for AD detection.

Four in vivo studies have revealed variable but significant changes in corneal nerve and cells among patients with AD compared to cognitively normal controls based on confocal microscopy and esthesiometry assessment. Örnek et al. reported a significantly lower corneal sensitivity and increased dendritic cell density in patients with AD compared to age- and sex-matched control patients [25]. Ponirakis also noted a stepwise decrease in corneal nerve fiber density, branch density, and fiber length between healthy controls, patients with mild cognitive impairment, and those with AD [26]. Similarly, Gundogan et al. found significantly lower corneal nerve fiber length, fiber density, branch density, and corneal sensitivity in patients with AD compared to controls [22]. In contrast, Dehghani et al. reported no significant differences in corneal nerve fiber length, fiber density, branch density, and diameter between AD and cognitively normal patients [27]. The discrepancy in findings could be due to the small, heterogenous patient cohorts and cross-sectional study designs.

Overall, these studies are limited by a relatively low sample size and cross-sectional nature, and may be confounded by the presence of other ocular and systemic conditions that impact corneal nerve fiber parameters. Observational cohort clinical trials are necessary to evaluate the effectiveness of changes in corneal nerve fiber parameters as diagnostic indicators of AD.

### 2.3. Iris/Pupillary Reaction

Prior research shows that pupillary reaction is associated with cognitive processing, including working memory span tasks [28]. This is due to the innervation of the pupillary sphincter muscle with parasympathetic neurons, including cholinergic neurons, and the muscle’s constriction in response to acetylcholine. Therefore, patients with AD may also present with changes in pupillary reaction and increased pupillary size.

Frost et al. reported that carriers with a mutation in the *APP* gene had a 75% longer recovery time to a bright flash of light than non-carriers [29]. Additionally, Frost et al. found that pupillary flash response (to white light stimuli) parameters classified AD with a relatively high sensitivity (84.1%), specificity (78.3%), and area under the curve (AUC) (89.6%) [30]. El Haj et al. demonstrated lower variability in pupil size during forward and backward memory span tasks, as well as during counting among patients with AD compared to controls [28]. Fotiou et al. found that pupillary light reflex parameters, including percentage recovery-redilation, latency, time for maximum velocity, time for maximum constriction, maximum constriction velocity, maximum constriction acceleration, amplitude, and ratio of baseline pupil diameter to the minimum pupil diameter after exposure to light stimuli after 3.5 s, were significantly lower among patients with AD compared to cognitively normal patients [31]. Prettyman et al. similarly demonstrated decreased resting pupil diameter, amplitude, and maximum dilational velocity of the darkness reflex in AD patients compared to age- and sex-matched control patients [32]. Lustig-Barzelay et al. developed a chromatic pupilloperimetry-based machine learning model which discriminated between patients at high risk for developing AD based on positive family history and cognitively normal patients. Transient pupil response latency demonstrated an area under the curve (AUC) of 0.90 ± 0.051 (left-eye) and 0.87 ± 0.048 (right-eye) in this discrimination task [33].

In contrast to the aforementioned studies on pupillary response, Kawasaki et al. found that patients with AD did not have a statistically significantly different pupillary contraction amplitude to red and blue light stimuli compared to control patients, and these study groups had a similar post-illumination pupillary response [34]. Similarly, Van Stavern et al. did not report any significantly different pupil flash response parameters between preclinical AD and normal-aging study groups [35]. It is possible that early-stage AD does not alter pupil responses, but further studies evaluating patients with preclinical AD are needed to confirm this.

Based on studies published to date, pupillary changes have the potential to serve as a noninvasive marker of AD pathology, but there are equivocal findings regarding the use of pupillary parameters in discriminating patients with and without AD. Further studies must be conducted to evaluate the diagnostic and prognostic accuracy of artificial intelligence algorithms for AD trained on pupillo-perimetry data.

### 2.4. Crystalline Lens

The crystalline lens has been identified as a site of neurodegenerative protein deposition in AD, and this process is thought to mimic insoluble protein deposition in cataract formation. Goldstein et al. reported that beta-amyloid protein was located in the cytosol of supranuclear/dense cortical lens fiber cells in patients with AD using slit lamp photomicroscopy, mass spectrometry, and immunohistochemistry [36]. Furthermore, patients with AD but not controls had equatorial supranuclear cataracts which showed birefringent Congo red staining, characteristic of amyloid protein [36]. In their later work, Moncaster et al. used ELISA to demonstrate increased accumulation of beta-amyloid in the lenses of patients with Down Syndrome, which results in three copies of the *APP* gene located on chromosome 21. This study also showed supranuclear opacification and beta-amyloid deposits in the cytosol of lens fiber cells [37]. Similarly, Kerbage et al. found that a fluorescent signature indicating the presence of a ligand bound to amyloid-β in the lens could discriminate probable AD patients from age-matched healthy patients with a relatively high sensitivity (85%) and specificity (95%) [38,39].

In contrast to the aforementioned studies, Michael et al. did not find Congo red staining or beta-amyloid immunohistochemical staining consistent with AD in lenses from postmortem donors with and without AD [40]. In later work, they confirmed an absence of amyloid-beta and tau in the postmortem lenses of AD donors using confocal Raman microspectroscopy [41]. Ho et al. also found negative immunostaining for beta-amyloid and phosphorylated tau, and negative Congo red staining in the postmortem eyes of AD patients [42]. Additionally, Williams et al. did not find any tau or beta-amyloid inclusions in the lens of patients with AD using immunohistochemistry [43].

Given the presence of lens opacities in the postmortem eyes of AD patients in some studies, Bei et al. evaluated whether cataract grade or lens opacities were associated with the risk of AD among patients with and without biomarkers for AD based on positron emission tomography and cerebrospinal fluid levels of Aβ42 [44]. While participants with positive biomarkers had more advanced cataracts and cortical light scattering compared to those with negative biomarkers, these differences were not statistically significant after adjusting for age [44]. Based on these results, the authors concluded that cataract grade and lens opacities could not be used to predict the risk of AD development [44].

Given the equivocal findings regarding the use of neurodegenerative protein deposition in the crystalline lens, further work is needed to determine why some lenses of patients with AD demonstrate beta-amyloid deposition while others do not. The variability in studies evaluating neuropathologic protein deposition in the lens could be due to differences in specimen preparation and staining, postmortem vs. in vivo examinations, time interval between death and specimen processing, and confounding lens opacities [45].

## 3. Posterior Segment

### 3.1. Retina

#### 3.1.1. Cell Layers

**Figure 2:**
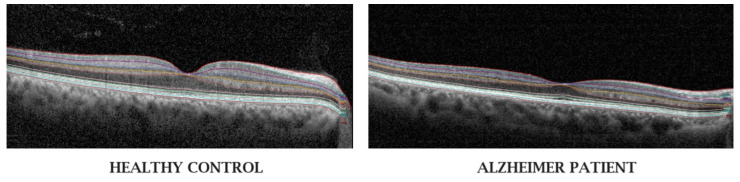
Optical coherence tomography of the retina of a healthy patient and that of a patient with Alzheimer’s disease from Garcia-Martin et al. [46]. Eyes of patients with AD have inner retinal layer thinning (involving the retinal nerve fiber layer, ganglion cell layer, and ganglion cell-inner plexiform layer) compared to those of healthy controls.

Optical coherence tomography (OCT) (Figure 2) [46] is a commonly used device to assess retinal tissue structure in clinical practice, and plays a central role in diagnosing and monitoring retinal disease progression and response to therapy in macular and optic nerve disorders. This non-invasive imaging technique captures cross-sectional, high-resolution images of the retinal cell layers at the macula and optic nerve. Given that these techniques allow for the analysis of changes in retinal thickness, blood flow velocity, and other structural parameters, they have been widely used in AD research.

There exist three main types of OCT. Time-domain OCT is one of the earliest forms of OCT, and is not widely used in clinical practice due to prolonged time for image acquisition, low axial resolution, and more recent technological advances to higher resolution [47] spectral domain-OCT (SD-OCT). SD-OCT detects the pattern of light reflected from each retinal cell layer, and is most frequently used in clinical practice today. Swept source-OCT (SS-OCT), which uses a tunable laser source, photodetector, and long-wavelength emission to capture images with greater resolution, is more expensive and mostly available to medical retina and vitreoretinal specialists in the research setting [47]. Furthermore, analysis of SS-OCT is limited by its lack of a normative database [47].

The impact of AD pathogenesis on the retina is multifactorial. Chronic activation of neuroinflammation decreases the integrity of blood-retinal barrier. Pro-inflammatory cytokines induce neuronal loss by damaging retinal ganglion cells and their axons [48]. Additionally, vascular insufficiency causes retinal ischemia, leading to cellular apoptosis [49,50]. Chronic gliosis of Müller cells leads to structural remodeling, including thinning of the inner retinal layers, which is detected by OCT imaging [51].

Meta-analyses of existing literature demonstrate inner retinal thinning in AD, particularly in the nerve fiber layer (RNFL), ganglion cell layer (GCL), and ganglion cell-inner plexiform complex (GC-IPL) [52,53]. These cell layers are likely affected by neurodegenerative disease processes because they are composed of nervous tissue [54]. The RNFL consists of axons (similar to white matter in the brain) of retinal ganglion cells (RGCs) while the GC-IPL consists of neuronal cell bodies and dendrites (similar to gray matter in the brain) of RGCs [54]. Thinning of the RNFL and GC-IPL occurs in all quadrants [55,56].

In contrast, Snyder et al. reported an increase in volume of the inner plexiform layer (IPL) among cognitively normal patients with positive family history and patient-reported memory deficits [57]. They also found that IPL volume positively correlated with the surface area of retinal inclusion bodies containing beta-amyloid [57]. They hypothesized that the IPL thickening could be due to decreased cholinergic activity in the IPL, which then causes beta-amyloid deposition, mimicking AD pathology in the neocortex [57]. Other studies have also reported deposition of AD neuropathologic proteins, including Aβ40, Aβ42, and pTau in the retina, with levels positively correlating with amyloid in the brain [58,59,60,61].

Given the extensive literature associating retinal structural changes with AD, OCT assessment of the retina may be used for diagnostic purposes for AD. Existing studies on artificial intelligence models trained on OCT data to distinguish patients with and without AD appear promising [62,63,64,65]. Therefore, patients with retinal thinning not explained by retinal pathology may be identified by their OCT findings and may benefit from neurocognitive workup for the early detection and symptomatic treatment of AD. Moreover, retinal conditions including diabetic retinopathy and retinal vein occlusions that are routinely monitored with OCT have been associated with an increased risk of AD [66,67].

Despite these promising studies, the clinical utility of OCT as a diagnostic tool for AD remains limited due to comorbid eye diseases, such as age-related macular degeneration, glaucoma, and diabetic retinopathy [68]. These ocular conditions can cause retinal thinning and vascular abnormalities that confound OCT interpretations in dementia [69,70,71]. These diseases are significantly associated with the presence of dementia [66], and affect similar demographic groups, which also limits the ability to isolate retinal changes secondary to dementia. Additionally, while OCT examination is relatively cheap and noninvasive for patients, the widespread implementation of this imaging modality in primary care or neurology clinics is limited by the relatively high cost of SD-OCT machines, which can range from $40,000 to $150,000 [72]. Additionally, there is no standardized protocol or consensus on retinal thickness thresholds for OCT-based AD diagnosis [73].

#### 3.1.2. Vasculature

**Figure 3:**
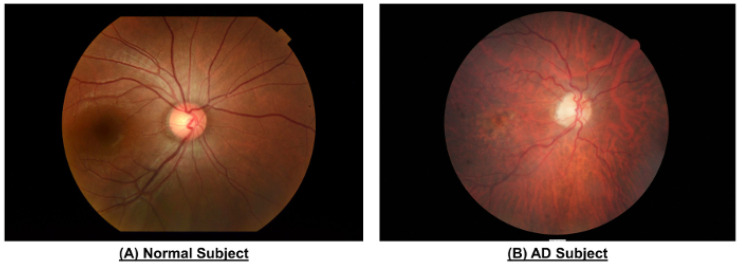
Color fundus photography of the eyes of patients with and without AD from Chan et al. [74]. Reproduced with permission from the Journal of Visualized Experiments. Those with AD demonstrate narrower vasculature, reduced vascular fractal dimensions, and greater vessel tortuosity.

The retinal microvasculature is similar in structure and function to cerebral small vessels, and therefore may parallel changes in cerebral vasculature in neurodegenerative disease processes [54]. Retinal vasculature is assessed with color fundus photography which is a non-invasive imaging technique commonly used in clinical practice to monitor disease progression. Fundus photography (Figure 3) [74] has been used to evaluate vascular parameters including caliber, fractal dimension, curvature tortuosity, number of first branching vessels, branching coefficients, junctional exponent deviation, and length-to-diameter ratios of retinal arterioles and venules in patients with dementia in research [75]. However, the analysis of imaging parameters is limited by the impact of magnification, refractive error, and variability in the quantitative measurements of retinal vascular parameters between software packages and within the same software package [75].

**Figure 4:**
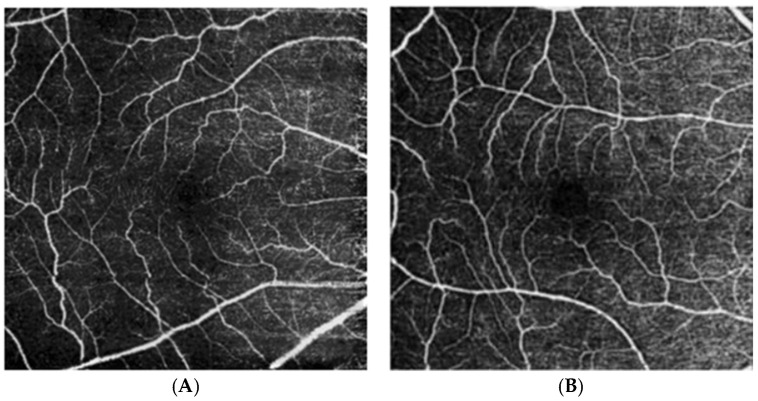
Optical coherence tomography angiography of the superficial vascular plexus of an eye from a cognitively unimpaired individual (**A**) and an individual with Alzheimer’s disease dementia (**B**) from Marquié et al. [76]. Those with AD demonstrate reduced vascular and perfusion density, as well as increased tortuosity.

Retinal vasculature can also be assessed with OCT angiography (OCTA) (Figure 4) [76], which is a more recent software addition to SD-OCT used to diagnose vascular conditions in the retina, optic nerve, and choroid [77,78]. Based on the detection of the speed of red blood cells flowing through vessels, retinal OCTA produces a 2-dimensional *en face* image of vascular density and perfusion of a specific tissue layer, such as the superficial and deep capillary plexuses [77,78]. From the patient perspective, this is a superior modality to fluorescein angiography, which requires an invasive intravenous injection of contrast material [77,78]. However, this modality is limited by artifacts from eye movements during image acquisition, confounding from media opacities, and projection artifacts from light reflected by the retinal pigment epithelium [77,78].

Systematic reviews and meta-analyses using OCTA and fundus photography indicate that retinal vessels in AD patients are lower in density and branching complexity, and may be narrowed or widened compared to cognitively normal patients [75,79]. Ma et al. reported that apolipoprotein E (*APOE*) ε4 carriers had a significantly lower perfusion density of the retina in the 6-mm Early Treatment of Diabetic Retinopathy Study circle and outer macular ring compared to noncarriers [80]. Li et al. found reduced vessel length density and vessel perfusion density in AD compared to control patients [81]. Cheung et al. found increased tortuosity of both retinal arterioles and venules in AD in a case–control study [82]. While most studies found no significant difference in the foveal avascular zone (FAZ) between patients with and without AD [83], O’ Bryhim et al. found a significantly greater FAZ in biomarker-positive preclinical AD compared to biomarker-negative patients [84]. Similarly, Bulut et al., Zabel et al., and Wu et al. reported a significantly greater FAZ in AD patients compared to controls [85,86,87]. This is likely due to variability in OCTA acquisition, including the scan size, layer definition, and artifact handling.

While existing literature points to differences in retinal vasculature parameters between AD and cognitively normal controls, further work is required to validate the software used to provide quantitative measurements from fundus photography. Given that digital fundus photography is a cost-effective imaging technique for screening that is preferred by patients and may increase compliance with scheduled visits [88], the use of retinal vascular parameters to distinguish patients with and without AD warrants further investigation after the refinement of software.

Recently, investigators have been increasingly interested in artificial intelligence approaches for the noninvasive identification of patients at risk for AD using ocular fundus imaging. Fundus-based AD screening is supported by epidemiologic studies demonstrating a link between retinal imaging features and incident dementia, such as Rebouças et al. [89]. This study found that retinal vascular caliber and tortuosity were significantly associated with mixed and vascular dementia in the Three-City-Alienor cohort [89]. A systematic review and meta-analysis of machine learning models trained with OCT, OCTA, and fundus photography to detect AD and Parkinson’s disease demonstrated moderate accuracy (AUC = 0.726) [90]. However, authors noted heterogeneity in the definition of early disease between studies and observational study designs as limitations, highlighting the gap between research use and implementation of AI systems in clinical practice. Wisely et al. developed a convolutional neural network trained on GC-IPL thickness maps, OCTA, ultra-widefield color fundus photography, and fundus autofluorescence images to detect AD [91]. They found that GC-IPL maps were the strongest inputs for higher diagnostic accuracy, and models trained with images only had a similar performance to models trained with imaging and patient data [91]. Similarly, Luengnaruemitchai et al. developed a convolutional neural network trained on color fundus photography that demonstrated a 96% accuracy, 99% sensitivity, and 90% specificity in discriminating between patients with and without AD and mild cognitive impairment [92].

If standardized protocols for OCT and OCTA-based AD diagnosis are established, AI-based risk stratification could be implemented in clinical practice. Changes in inner retinal thickness and vascular density could be used to flag patients of ophthalmology clinics who may be at high-risk for developing AD. These patients can then be referred to their primary care provider for a neurology referral and cognitive assessment.

### 3.2. Optic Nerve

The optic nerve has a direct anatomical and functional connection to the central nervous system, and can be assessed with similar neuroimaging modalities used to examine the brain in AD, including positron emission technology and magnetic resonance imaging. These techniques allow for high-resolution visualization and quantification of structural and metabolic brain changes associated with AD. However, they are costly, involve long image acquisition times, and may not be available in low-resource areas. Non-invasive and cheaper alternatives to these techniques that have also been used to assess disc parameters in AD include computed tomography (CT) and OCT of the optic nerve head.

Wu et al. compared the CT density and beta-amyloid level of the intraorbital optic nerve using CT imaging and ^18^F-flutemetamol amyloid positron emission tomography, respectively [93]. They found that the intraorbital optic nerve standardized uptake value ratio was significantly greater and optic nerve density was significantly lower in AD patients compared to cognitively normal patients [93]. Additionally, these optic nerve parameters were able to distinguish AD from mild cognitive impairment (MCI) and MCI from cognitively normal patients with an AUC of 0.9167 and 0.8951, respectively [93].

Changes in the optic nerve head have also been associated with AD severity and duration. Tsai et al. reported that higher pallor area-to-disc ratios were associated with higher Alzheimer’s Disease Assessment Scale scores and duration of disease using fundus photography and an optic nerve head analyzer [94]. Additionally, patients with AD had a significantly increased cup-to-disc ratio and cup volume and decreased disc rim area compared to age- and race-matched cognitively normal control patients [94]. In contrast, Kurna et al. reported no significant difference in the rim area, rim volume, linear cup-to-disc ratio, height variation contour, and cup shape measure between AD and control patients using confocal scanning laser tomography [95].

Peripapillary nerve fiber loss is hypothesized to be associated with neurodegenerative conditions including AD [95]. Several studies reported thinning of the peripapillary RNFL in AD, particularly in the superior and inferior quadrants [96,97,98]. However, Gharbiya et al. and van de Kreeke et al. found no significant difference in the peripapillary RNFL between patients with and without AD and preclinical AD, respectively [99,100].

Using magnetic resonance imaging and in vivo volumetry, Kusbeci et al. reported significantly lower optic nerve volume in AD compared to cognitively healthy participants [101]. However, Davies et al. found no significant difference in the mean cross-sectional area of the optic nerve, myelinated axon surface density, total axon number, or mean cross-sectional axon area in the postmortem intraorbital optic nerve segments of patients with AD compared to controls [102]. The postmortem specimen in Davies et al. may have been susceptible to atrophy and decreased axonal integrity following death.

Cesareo et al. reported glaucoma-like findings among patients with AD, which may be related to an underlying association between glaucoma and AD [66,103]. Despite a lower intraocular pressure, those with AD had a worse mean deviation, pattern standard deviation, and altered Glaucoma Hemifield Test compared to age- and sex-matched controls. These measures are used in clinical practice to diagnose and monitor glaucoma progression, suggesting their utility in AD detection as well.

Optic nerve pallor may be a proxy for axonal loss, which can be quantified using colorimetric analysis of fundus photography [104]. Salobrar-García et al. reported no significant difference in the hemoglobin levels of the optic nerve head tissue using a postmortem analysis [105]. In contrast, Bambo et al. reported that mean hemoglobin percentage and hemoglobin content in the neuroretinal rim were reduced in AD compared to age- and sex-matched controls using fundus photography and colorimetric assessment [104]. Axonal loss at the optic nerve head in AD can also be confirmed with histological staining. Syed et al. reported decreased axonal density at the central and peripheral optic nerve with loss of smaller-sized axons in AD compared to control patients [106].

These studies highlight the growing interest in the optic nerve as a potential ocular biomarker for AD diagnosis. While prior studies demonstrate structural and functional changes in the optic nerve head, peripapillary nerve fiber layer, and infraorbital nerve volume in AD, there is variability across imaging techniques that may be due to differences in resolution, sample size, disease severity, and methodology. Among the imaging modalities used to assess the optic nerve, OCT is the most widely used and seems the most promising in serving a non-invasive diagnostic imaging technique. Future studies could further explore the clinical utility of optic nerve imaging in AD diagnosis with standardized protocols and larger, diverse cohorts.

### 3.3. Choroid

Similarly to the retina, the choroid demonstrates morphological and vascular changes in AD. The choriocapillaris provides oxygen to the outer retina and vitreous chamber [107]. Choroidal ischemia is linked to changes in choroidal thickness, and these parameters are often abnormal in retinal degenerative conditions including age-related macular degeneration. Changes in the choroidal vasculature and blood flow are hypothesized to mimic cerebrovascular changes in AD [107]. Choroidal structure and vasculature can be assessed with SS-OCT and OCTA.

Choroidal thinning in AD has been established in several studies [53,81,99,105,107,108,109,110,111]. Li et al. also found that decreased subfoveal choroidal thickness was predictive of AD [107] in an in vivo analysis of 71 patients using enhanced depth imaging OCT. In contrast, Asanad et al. reported choroidal thickening in the central macula using histopathological techniques in postmortem AD compared to controls and this thickening was associated with stromal vessel number [112]. Similarly to Asanad et al., Castilla-Martí et al. demonstrated choroidal thickening in the inner nasal and inferior macula, as well as the outer temporal, nasal, and inferior macula in AD compared to cognitively normal controls [113].

Corradetti et al. reported increased choriocapillaris flow deficits in patients with presymptomatic AD compared to healthy patients [114]. While there was no difference in the choroidal vascularity index, total choroidal area and luminal area were significantly higher in AD in Robbins et al. [109]. In contrast, Ma et al. reported no significant differences in total choroidal area, luminal area, or choroidal vascularity index between *APOE* ε4 allele carriers and non-carriers [80]. Kwapong et al. reported that a lower choriocapillaris density on SS-OCT angiography (SS-OCTA) was associated with increased neuropathologic proteins in AD including Aβ40, Aβ42, and pTau181 [115]. Burke et al. found that increased total choroidal area and vessel area was associated with the presence of *APOE* ε4 allele or a positive family history for AD [116]. Di Pippo et al. found a significantly lower choriocapillaris flow area in AD compared to controls [117].

Collectively, these studies demonstrate that the choroid undergoes structural changes in AD that mirror pathologic changes in the retina and the brain. Choroidal thinning is the most consistently-reported finding, and may serve as a potential biomarker for AD diagnosis similar to inner retinal thinning. However, there is equivocal evidence in changes in vascular parameters, and the variability in findings may be attributed to genetic and systemic factors such as *APOE* ε4 status, as well as eye diseases that can impact choroidal thickness including high myopia (thinner choroid) or central serous chorioretinopathy (thicker choroid). The association of choriocapillaris parameters with AD-specific proteins including beta-amyloid strengthens the association between ocular vasculature and AD pathophysiology.

## 4. Ocular Fluids

In addition to central nervous system imaging, quantification of neuropathologic proteins in CSF serves as the gold standard for underlying AD pathology detection [118]. However, CSF sample collection involves a lumbar puncture, which is an invasive procedure. The routine use of CSF analysis is also limited by issues with transportation, storage, and cost [119]. Similar to the detection of AD neuropathologic proteins in CSF, emerging studies have identified the presence of beta amyloid and tau proteins in ocular fluids. In fact, Sampani et al. measured relative concentrations of Aβ40, Aβ42, total Tau, ptau181, glial fibrillary acidic protein (GFAP), and neurofilament light chain (NfL), and found that concentrations were higher in eye fluids including tear secretions, aqueous humor, and vitreous humor, compared to plasma [120].

### 4.1. Tear Secretions

In contrast to vitreous and aqueous humor collection, tear fluid biomarkers can be sampled non-invasively, and therefore, there is a particular interest in the use of tear secretions in AD diagnosis. Tear fluids are most commonly collected via Schirmer strips, which involve placing the strip in the lower cul-de-sac, lower conjunctival fornix, external canthus of eyelids, or lower conjunctival sacs for up to 5 min, and capillary collection, which involves placing microcapillary tubes into the ventral cul-de-sac or lacrimal lake at the inner or external canthi for up to 5 min [121]. While Schirmer strips are inexpensive and simple to use, their use in AD research is limited by a low volume of tears collected, evaporation [122], and by the inclusion of highly abundant proteins from the ocular surface epithelium that may interfere with neuropathologic protein analysis [121].

While Kalló et al. reported that participants with AD had a significantly higher protein concentration and flow rate compared to controls with capillary collection, Kenny et al. reported no significant differences in these parameters between study groups with Schirmer strips [123,124]. Additionally, Kalló et al. reported that the combination of lipocalin-1, dermcidin, lysozyme-C, and lacritin in tears was diagnostic of AD with a sensitivity of 81% and specificity of 77% [123]. Örnek et al. found decreased tear break-up time and Schirmer’s 1 test scores in AD compared to controls [25].

Gijs et al. found that AD participants had relatively higher levels of Aβ38, Aβ40, Aβ42, and total Tau compared to healthy controls [125]. In contrast, Gharbiya et al. found significantly lower Aβ42 levels in tears of AD participants compared to healthy controls, and hypothesized that this could parallel the lower Aβ42 in CSF due to sequestration in the brain [126]. They also hypothesized that beta-amyloid could be sequestered in lacrimal gland cells in AD participants [126]. While the aforementioned studies directly compared AD participants with healthy controls, Del Prete reported the presence of Aβ42 in tear samples of two cognitively normal participants with a family history of AD, and the absence of this neurodegeneration biomarker in one healthy participant with no family history [127].

Kenny et al. reported increased levels of microRNA among participants with AD, likely due to the cellular disruption and neurodegeneration that characterize AD [124]. They particularly found that microRNA-200b-5p was significantly elevated in the tear fluid of AD participants, which has not been previously reported, and is associated with reducing cognitive impairment via insulin signaling in murine models [128]. Interestingly, Kenny et al. also detected elF4E, a translation initiation factor involved in protein synthesis, in tear samples among AD participants, but not among those of healthy controls [124].

In summary, tear fluid analysis represents a potentially emerging diagnostic method for AD, offering non-invasive, accessible, and scalable neuropathologic protein detection. Existing literature shows that differences in proteins, including AD-specific neuropathologic proteins, and microRNAs can be used to distinguish patients with and without AD. The detection of beta-amyloid in tears of cognitively normal patients with a positive family history raises the potential for early detection and risk stratification. However, the variability in results and tear collection methods, as well as limitations in tear collection methods require further investigation before tears can be used to diagnose AD in clinical practice.

In clinical practice, translation of tear-based biomarkers could be appropriate in primary care, neurology, geriatric, and optometry or ophthalmology settings. Given that tear sampling is relatively quick, cheap, and noninvasive, this could potentially be offered routinely to high-risk older patients with a positive family history for AD. Prior to implementation, further research is needed to standardize assays for measuring tear biomarkers, establish diagnostic windows for normal vs. pathologic levels, and determine the sensitivity and specificity of these windows for detecting neurodegenerative diseases. After these steps, assay results for each protein could be combined into high-, intermediate-, or low-risk categories based on pre-defined thresholds. Patients in the high- and intermediate-risk categories could then be referred to neurology for a full cognitive assessment for potential AD or other dementia.

### 4.2. Aqueous Humor

Aqueous humor is the circulating and regenerative watery fluid found in the anterior chamber of the eye, secreted by the ciliary body and absorbed into the trabecular meshwork [129]. This fluid regulates the intraocular volume and pressure, and pathology involving variations in intraocular pressure includes ocular hypertension and glaucoma [129]. Given that glaucoma is associated with AD [66] and that glaucoma and AD share retinal structural changes including ganglion cell thinning, aqueous humor may have a potential role in AD diagnosis. However, aqueous humor collection requires anterior chamber paracentesis, which is an invasive procedure and has potential for complications, such as damage to the iris and lens, as well as endophthalmitis and corneal abscess [130].

There are limited studies demonstrating the presence of AD neuropathologic proteins in aqueous humor. Inoue et al. reported that the aqueous humor of patients with primary open-angle glaucoma had significantly higher levels of apolipoprotein (Apo) AI, ApoCIII, ApoE, and α2-macroglobulin compared to that of patients with cataracts [131]. However, Cappelli et al. did not find significantly different levels of Aβ42 in the aqueous humor of patients with glaucoma and that of control patients [132]. Similarly, Janciauskiene et al. found no significant differences in the levels of Aß38, Aß40, or Aß42 between patients with cataracts alone, cataract and glaucoma, cataract and pseudoexfoliation syndrome, and cataract and diabetic retinopathy [133].

Patel et al. reported that pTau181 in the aqueous humor was positively correlated with plasma pTau181, and negatively correlated with Montreal Cognitive Assessment scores (commonly used in dementia evaluation) among patients who were not previously diagnosed with cognitive impairment [134]. Bai et al. detected NfL, Aβ40, Aβ42, GFAP, and pTau181 in aqueous humor samples of patients with neovascular age-related macular degeneration (nAMD) or senile cataracts. NfL levels in the aqueous humor of patients with nAMD were negatively correlated with lower Mini-Mental State Examination (MMSE) scores [135].

Although aqueous humor is not as easily accessible as tear secretions, existing literature suggests that this fluid contains AD-specific neuropathologic proteins, particularly among patients with glaucoma. Shared structural retinal changes justify the exploration of the diagnostic role of aqueous humor in AD. While some studies demonstrate beta-amyloid, hyperphosphorylated tau, and neurofilament light chain in the aqueous humor, the results are overall inconsistent with other reports finding no significant difference between patients with and without AD. However, the correlation between hyperphosphorylated tau and cognitive scores in patients without known dementia indicates the utility of aqueous humor analysis in early or preclinical detection. That being said, the invasive nature of aqueous humor collection, coupled with its potential complications, limits the clinical utility of aqueous humor in routine AD diagnosis compared to other techniques such as OCT and OCTA.

### 4.3. Vitreous Humor

Vitreous humor is a gel-like, non-regenerative, embryological remnant fluid that fills the posterior chamber of the eye and helps maintain the structure of the globe [136]. This fluid is involved in pathology including vitreous hemorrhage and posterior vitreous detachment. Prior studies have evaluated the vitreous humor for neurodegenerative biomarkers given the association between retinal pathology, including diabetic retinopathy and age-related macular degeneration, and AD [66]. Similarly to aqueous humor collection, vitreous humor collection requires an invasive procedure (pars plana vitrectomy). However, vitreous humor may be of interest in AD research because this fluid is isolated from systemic circulation and therefore may reflect both retinal and central nervous system pathology.

To our knowledge, there exists one study evaluating vitreous neuropathologic proteins in patients with AD compared to cognitively normal participants. Vig et al. reported significantly higher total Tau levels in postmortem vitreous humor in those with pathologically confirmed AD compared to controls [137]. Other studies have indirectly evaluated the association between AD-associated biomarkers in vitreous humor and neurocognitive defects. Wright et al. reported that lower levels of Aβ40, Aβ42, and total Tau were significantly associated with lower MMSE scores in cognitively normal participants [138]. While Subramanian et al. did not find any associations between *APOE* ε2 or ε4 genotypes and vitreous NfL, they did report vitreous NfL levels to be positively correlated with vitreous Aβ40, Aβ42, and total Tau [139].

The limited evidence evaluating the diagnostic potential of vitreous humor in AD shows that the vitreous has elevated neuropathologic proteins in confirmed postmortem AD patients, and these proteins correlate to cognitive scores in healthy living patients. Furthermore, neurodegenerative proteins are present at a higher concentration in vitreous humor than plasma [120]. While these studies indicate the potential involvement of vitreous humor in AD detection, vitreous humor collection is invasive and may only be indicated among patients undergoing standard-of-care vitrectomy for retinal pathology. Patients incidentally found to have elevated neuropathologic biomarkers may benefit from closer monitoring for cognitive pathology.

## 5. Plasma Biomarkers

While ocular imaging and fluid biomarkers, particularly OCT-based markers, offer a noninvasive option for AD diagnosis, plasma biomarkers are currently closer to clinical implementation for AD diagnosis. Plasma levels of phosphorylated tau, beta-amyloid, and NfL have shown strong associations with the presence of AD [140,141,142]. Plasma assays for neuropathologic proteins are relatively low-cost, minimally invasive, and scalable for use in primary care, as specimens may be collected during routine blood testing, making these assays feasible for population-level screening [143]. However, there exist several barriers to implementation in clinical practice. Patient factors, including comorbidities, medications, and demographics, can confound the concentration of neuropathologic proteins [144]. Reference ranges need to be adjusted for these factors. Furthermore, the recommended two-cutoff thresholding (which defines positive, intermediate, and negative) categories based on beta-amyloid creates an indeterminate (intermediate) category which can require further confirmatory testing with PET and CSF, delaying referral for further cognitive testing [144]. Additionally, pre-analytical sample handling involves steps that must be performed precisely [145]. Details such as the correct primary collection tube type, delays before centrifugation and freezing, and storage temperature have been shown to impact beta-amyloid protein levels, and may be difficult to control in a real-world setting [145]. Beyond this, there exists analytical variability across platforms, which limits the comparability between assays in the absence of standardized reference ranges that can be applied to multiple platforms.

In contrast, the implementation of ocular imaging techniques such as OCT and OCTA in primary care and neurology practices is limited by a high machine cost and need for trained personnel [72]. Moreover, co-existing ocular conditions as well as the absence of standardized diagnostic thresholds or protocols also limit their clinical utility [68]. While individuals with eye diseases are more likely to undergo ocular imaging than those without ocular conditions, population-level screening with blood-based biomarkers in primary care settings would capture those with and without eye disease. Overall, ocular imaging-based biomarkers may serve as adjunct diagnostic tools, but plasma biomarkers are currently more feasible for implementation in clinical practice.

## 6. Conclusions

In conclusion, the shared embryological origins between the eye and the brain may result in shared pathophysiological ocular and neurological changes in AD. This is evident by the deposition of AD neuropathologic proteins throughout the anterior and posterior segments of the eye. Imaging modalities including OCT and OCTA most strongly demonstrate the presence of AD biomarkers in retinal cell layers and vasculature. AD neuropathologic proteins are detectable in ocular fluids, including tear secretions, aqueous humor, and vitreous humor, but warrant further research into their diagnostic and predictive ability. However, some fluids, including aqueous and vitreous humor, involve invasive collection procedures that limit their utility in clinical practice for AD diagnosis. Overall, ocular biomarkers for AD represent a relatively less invasive and potentially cheaper modality for diagnosing AD. Based on the sources of discrepancies between studies evaluating similar associations between ocular structures and AD, future research should establish and implement protocols for commonly used imaging techniques including OCT and OCTA. These protocols should specify scan size, slab definitions, artifact handling, and minimum quality thresholds to ensure comparable data between studies. Furthermore, given the limitations of Schirmer strips, future studies should use the capillary collection technique for higher-quality analysis of tear secretions. When possible, research should pair in-vivo slit-lamp and fluorescent ligand imaging with postmortem assays among the same donors, with a longitudinal study design and standardized time-to-fixation, staining, and other processing steps to better establish the presence of neuropathologic proteins in the lens. Overall, research evaluating the association between eye and AD is limited by cross-sectional study designs and small cohorts. Longitudinal, prospective studies with larger sample sizes would be more appropriate in establishing the causal and temporal relationships required to understand the underlying pathophysiology driving changes in the eye and brain during neurodegenerative disease development. Future studies could evaluate the performance of artificial intelligence models trained on multimodal ophthalmic information in diagnosing AD.

## Figures and Tables

**Table 1:** Associations Between Ocular Tissue and AD.

Ocular Tissue	Methodology	Changes in Ocular Structure/Function Reported Among AD Participants Relative to Controls
Anterior Segment		
Conjunctiva	Computer-assisted intravital microscopy of conjunctival microcirculation	• Higher whole-blood viscosity and greater vasculopathy
Cornea	In vivo confocal microscopy and esthesiometry	• Lower corneal sensitivity • Reduced corneal nerve fiber length, density, and branching • Increased dendritic cell density
Iris/Pupillary Reaction	Pupillary light/flash response testing, chromatic pupilloperimetry with machine learning	• Longer recovery to bright flash in APP-mutation carriers • Lower variability in pupil size during memory/counting tasks • Reduction in light reflex parameters
Crystalline Lens	Slit-lamp photomicroscopy, mass spectrometry, immunohistochemistry, ELISA, fluorescent ligand–based detection	• β-amyloid detected in supranuclear/dense cortical lens fibers • Congo-red staining of supranuclear cataracts • Fluorescent ligand signature discriminated probable AD from controls (reported sensitivity 85%, specificity 95%)
Posterior Segment		
Retinal Layers	Optical coherence tomography (OCT)	• Inner retinal thinning (retinal nerve fiber layer, ganglion cell layer, ganglion cell-inner plexiform layer complex) • Increased inner plexiform layer volume due to β-amyloid accumulation
Retinal Vasculature	Fundus photography, OCT angiography (OCTA)	• Lower vessel density and branching complexity • Increased arteriolar/venular tortuosity and increased foveal avascular zone • Lower macular perfusion in *APOE* ε4 carriers
Optic Nerve	Computed tomography (CT), intraorbital amyloid positron emission tomography (PET), optical coherence tomography of the optic nerve head, magnetic resonance imaging (MRI)	• Higher intraorbital optic-nerve amyloid PET uptake and lower CT density • Increased cup-to-disc ratio and decreased rim area, increased pallor • Thinning of peripapillary retinal nerve fiber layer • Lower optic-nerve volume on MRI • Glaucoma-like visual field test deviations • Reduced rim hemoglobin
Choroid	OCT, OCTA	• Choroidal thinning • Increased choriocapillaris flow deficits • Lower choriocapillaris density due to neuropathologic protein accumulation
Ocular Fluids		
Tear Secretions	Schirmer strips, capillary tubes	• Higher protein concentration and flow rate • Shorter tear break up time and lower Schirmer scores • Higher β-amyloid (Aβ38, Aβ40, and Aβ42) and total tau levels • Increased microRNA-200b-5p and detectable elF4E (translation initiation factor)
Aqueous Humor	Anterior chamber paracentesis	• pTau181 in the aqueous humor positively correlates with plasma pTau181 • pTau181 and neurofilament light chain negatively correlate with Montreal Cognitive Assessment scores and Mini-Mental State Examination (MMSE) scores
Vitreous Humor	Pars plana vitrectomy	• Higher vitreous total tau • Lower vitreous Aβ40, Aβ42, and total Tau levels associated with lower MMSE scores (among cognitively normal individuals) • Vitreous neurofilament light chain positively correlates with vitreous Aβ40, Aβ42, and total Tau

## Data Availability

No new data were created or analyzed in this study.

## Abbreviations

The following abbreviations are used in this manuscript:

AD	Alzheimer’s disease
*APOE*	Apolipoprotein E
APP	Amyloid precursor protein
AUC	Area under the curve
CSF	Cerebrospinal fluid
CT	Computed tomography
GC-IPL	Ganglion cell-inner plexiform layer
GCL	Ganglion cell layer
GFAP	Glial fibrillary acidic protein
IPL	Inner plexiform layer
MCI	Mild cognitive impairment
MMSE	Mini-Mental State Examination
NfL	Neurofilament light chain
OCT	Optical coherence tomography
OCTA	Optical coherence tomography angiography
pTau	Hyperphosphorylated tau
RGC	Retinal ganglion cell
RNFL	Retinal nerve fiber layer
SD-OCT	Spectral domain optical coherence tomography
SS-OCT	Swept source optical coherence tomography
SS-OCTA	Swept source optical coherence tomography angiography
